# Influence of light alcohol consumption on lifestyle-related diseases: a predictor of fatty liver with liver enzyme elevation in Japanese females with metabolic syndrome

**DOI:** 10.1186/s12876-016-0431-6

**Published:** 2016-02-18

**Authors:** Masahiro Sogabe, Toshiya Okahisa, Tadahiko Nakagawa, Hiroshi Fukuno, Masahiko Nakasono, Tetsu Tomonari, Takahiro Tanaka, Hironori Tanaka, Tatsuya Taniguchi, Naoki Muguruma, Tetsuji Takayama

**Affiliations:** Department of General Medicine and Community Health Science, Institute of Biomedical Sciences, Tokushima University Graduate School, 3-18-15 Kuramoto-cho, Tokushima City, Tokushima 770-8503 Japan; Department of Gastroenterology and Oncology, Institute of Biomedical Sciences, Tokushima University Graduate School, Tokushima, Japan; Department of Gastroenterology, Kagawa Prefectural Cancer Detection Center, Takamatsu, Japan; Department of Internal Medicine, Higashi Tokushima Medical Center, Tokushima, Japan; Department of Internal Medicine, Tsurugi Municipal Handa Hospital, Tokushima, Japan

**Keywords:** Light alcohol consumption, Fatty liver with ALT elevation, Females, Metabolic syndrome

## Abstract

**Background:**

Although heavy drinking is known to lead to liver injury, some recent studies have reported that light alcohol consumption (LAC) may play a protective role against fatty liver in the general population, and may even play a protective role against non-alcoholic fatty liver disease (NAFLD) in males with metabolic syndrome (MS). However, the association between LAC and fatty liver with liver enzyme elevation in females with MS is unclear.

**Methods:**

Participants of this study were 20,853 females who underwent a regular health check-up between April 2008 and March 2012 at our hospital. Enrolled subjects were 1141 females with MS, who underwent all necessary tests and drank less than 20 g/day of alcohol. We investigated the presence of fatty liver with liver enzyme elevation, defined in this study as alanine aminotransferase (ALT) levels ≧31 IU/I, and the association between LAC and fatty liver with ALT elevation.

**Results:**

There was no significant difference in the prevalence of fatty liver and ALT between light drinkers and non-drinkers. The prevalence of individuals receiving a treatment for dyslipidemia and impaired glucose tolerance (IGT) was significantly lower in light drinkers than in non-drinkers. Body mass index (BMI), waist circumference (WC), diastolic blood pressure (DBP), triglyceride (TG), uric acid (UA), IGT, and visceral fat type MS (V-type MS) were significant predictors of the prevalence of fatty liver with ALT elevation in logistic regression analysis. The odds ratio [OR] (95 % confidence interval [CI], *p* value) for fatty liver with ALT elevation were as follows: BMI, 2.181 (1.445–3.293, *p* <0.001); WC, 1.853 (1.280–2.684, *p* <0.01); DBP, 1.604 (1.120–2.298, *p* <0.05); TG, 2.202 (1.562–3.105, *p* <0.001); UA, 2.959 (1.537–5.698, *p* <0.01); IGT, 1.692 (1.143–2.506, *p* <0.01); and V-type MS, 3.708 (2.529–5.437, *p* <0.001).

**Conclusions:**

There was no significant difference in the prevalence of fatty liver with ALT elevation in females with MS between light drinkers and non-drinkers, suggesting that other factors such as BMI, WC, V-type MS, and lifestyle-related disease may be more important than LAC for the prevalence of fatty liver with ALT elevation.

## Background

The prevalence of MS defined as obese individuals with abnormalities in hypertension, glucose metabolism, and dyslipidemia, has been increasing in countries of both advanced and emerging economies. This increase in prevalence is problematic due to the association of MS and various other diseases, as well as an increase medical expenses. Additionally, in Asian and western countries, the prevalence of non-alcoholic fatty liver disease (NAFLD), which is strongly associated with MS, has also been increasing [1, 2]. Although heavy drinking is believed to lead to liver injury [3], several recent studies have reported that moderate or light drinking plays a protective role against fatty liver [4–8]. Drinking may be problematic for persons with MS due to the differences in clinical background between persons with and without MS and the increase in total caloric intake due to drinking. Although light alcohol consumption (LAC) was shown to play a protective role against NAFLD in males with MS in a recent study [9], the prevalence of MS and NAFLD, and the influence of alcohol consumption are known to differ markedly between males and females. The present study investigated the association between LAC and fatty liver with alanine aminotransferase (ALT) elevation in females with MS.

## Methods

### Subjects and study design

Subjects were 20,853 females (mean age ± standard deviation 49.2 ± 11.5 years range 17–88), residing around Takamatsu city, who underwent a regular health check-up at our hospital between April 2008 and March 2012. We excluded subjects who did not fulfill the diagnostic criteria for MS, who were positive for hepatitis B surface antigen (HBsAg) and/or hepatitis C antibody (HCVAb), who did not hope to undergo ultrasound examination, or who consumed 20 g/day or more of alcohol. All subjects were informed that their clinical data may be retrospectively analyzed for an epidemiological study, and informed consent was obtained. This study was a cross-sectional study elucidating the association between LAC and fatty liver with ALT elevation in females with MS. The study design was approved by the Ethics Committees of Kagawa Prefectural Cancer Detection Center, and the study was performed in conformity with the Declaration of Helsinki.

### Physical examination and serum biochemistry

Height and body weight were obtained from the participants. Height was measured to the nearest 0.1 cm and body weight was measured to the nearest 0.1 kg. Body mass index (BMI) was calculated as the weight (in kilograms) divided by the square of the height (in meters). Waist circumference (WC) was measured at the umbilical level. Venous blood samples were taken from all subjects following a 12-h overnight fast. Aspartate aminotransferase (AST), ALT, gamma-glutamyl transpeptidase (GGT), total cholesterol (T-CHO), high-density lipoprotein (HDL) cholesterol, triglycerides (TG), low-density lipoprotein (LDL) cholesterol, uric acid (UA), fasting plasma glucose (FPG), and hemoglobin A1c (HbA1c) (National Glycohemoglobin Standardization Program [NGSP]) were analyzed immediately by common enzymatic methods using an auto analyzer (TBA-80FR; Toshiba Medical System Tokyo, Japan).

### Evaluation of alcohol consumption

Lifestyle-related information such as medical history, smoking status, and alcohol consumption were obtained by a common standardized self-response questionnaire. The amount of alcohol consumed per drinking day was calculated in grams using representative percent alcohol by volume for each type of alcoholic beverages: 5 % for beer, 10 % for wine, 16 % for Japanese sake, 25 % for shochu, and 34 % for whiskey. Subjects were divided into two categories by the drinking information: non-drinkers, those drinking 12 drinks (one drink means less 10 g) or less per year of less than one drink/drinking day; and light drinkers those drinking, from more than zero to less than 20 g/drinking day. In addition, light drinkers were divided into two subcategories: minimal drinkers, those drinking from more than zero to less than 10 g/drinking day; and very light drinkers, those drinking from 10 g to less than 20 g/drinking day.

### Diagnosis of MS

There are several diagnostic criteria for MS. We used the diagnostic criteria for MS proposed by the International Diabetes Federation (IDF) because this criteria has been used worldwide [10]. The MS criteria used in this study were as follows: WC must exceed 80 cm, and two or more of the following components must be present: (1) hypertension: blood pressure ≥130/85 mmHg or medicated for hypertension (2) dyslipidemia: HDL-C <50 mg/dl and/or TG ≥150 mg/dl, or medicated for dyslipidemia (3) impaired glucose tolerance: FPG ≥100 mg/dl or medicated for diabetes.

### Ultrasonography

Standard ultrasonography was performed with the subjects to assess the abdomen including the liver, gallbladder, pancreas, kidney, and spleen, in a morning fasting state by trained technicians. A Xario SSA-660A instrument was used with a 3.5 MHz convex-array probe (Toshiba Medical System, Tokyo, Japan) for ultrasonography. During ultrasound examination, more than 20 abdominal images were saved in the database and reviewed by gastroenterologists, who had more than 10 years of experience in ultrasonography, without knowledge of the results of the subject’s data, such as blood-test screening. We defined fatty liver by ultrasonography by the presence of liver-kidney echo contrast and liver brightness, as well as having deep attenuation and/or liver vessel blurring [11]. A liver biopsy is known to be the gold standard for the diagnosis of NAFLD. However, liver biopsy is not realistic to perform in a medical check-up because this method has a risk of bleeding. Thus, we investigated fatty liver with ALT elevation in the present study. Alanine aminotransferase elevation was defined as an ALT level of ≥31 IU/l in this study. Also, visceral fat was assessed by the abdominal wall fat index (AFI), defined by the ratio of preperitoneal and subcutaneous fat thicknesses [12]. This procedure has been widely employed in medical check-ups for the assessment of visceral fat due to the significant correlation between the ratio of visceral and subcutaneous fat area by computed tomography (CT) and AFI by ultrasonography [12]. Although CT is the best method for assessment of the visceral fat, radiation exposure by CT is a risk for young females. Therefore, we selected the use of ultrasonography to assess visceral fat in the present study.

### Statistical analysis

This study was a cross-sectional study investigating the association between LAC and fatty liver with ALT elevation in females with MS. Baseline data are expressed as means ± standard deviation (SD) for all continuous variables and as subject number (%) for categorical variables. All statistical differences were considered significant at a *P* value of less than 0.05. The *χ*2-test or Student’s t-test was used to compare and determine differences between two groups for independent samples and the *P* value was adjusted for confounding variables using analysis of covariance. Factors with a significant influence on the prevalence of fatty liver with ALT elevation were then determined by univariate analysis. Age, BMI, WC, SBP, DBP, TG, HDL-C, UA, IGT, MS type, smoking status, and drinking status were then subjected to a multivariate logistic regression analysis. Results are presented as odds ratios (ORs) with 95 % confidence interval (CI) for each variable. All statistical analyses were performed using Med Calc Software (Broekstraat, Mariakerke, Belgium).

## Results

### Enrollment

**Fig. 1:**
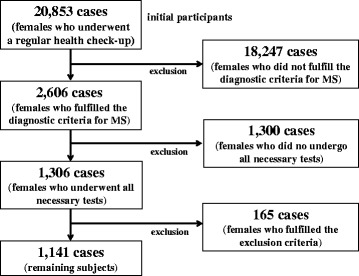
Flow diagram of subjects included and excluded from the present study. MS = metabolic syndrome

Enrolled subjects are shown in Fig. 1. Initial participants were 20,853 females who underwent a regular health check-up between April 2008 and March 2012 at our hospital. Subjects were enrolled after application of the inclusion and exclusion criteria described in the Methods section (Subjects and study design). Of the 20,853 subjects, 2606 (12.5 %) females fulfilled the diagnostic criteria for MS. Of the 2606 subjects, 1306 subjects answered the self-response questionnaire, underwent physical examinations, blood-test screening, and abdominal ultrasonography. Of the 1306 subjects, we excluded 165 subjects who fulfilled the exclusion criteria, and the remaining 1141 subjects were enrolled in this study.

### Subject characteristics

The characteristics of enrolled subjects are shown in Table 1. Mean age BMI, and WC was 56.6 ± 8.3 years (range, 29–85), 26.3 ± 3.6 kg/m^2^ (range, 18.5–50.8), and 90.0 ± 7.2 cm (range, 80–117.5), respectively. Prevalence of hypertension, dyslipidemia, and impaired glucose tolerance (IGT) was 82.1, 93.4, and 74.7 %, respectively. The prevalence of fatty liver in all subjects was 56.3 %. Of the 1141 subjects with MS, the number of subjects with visceral type MS (V-type MS) and subcutaneous type MS (S-type MS) was 170 (14.9 %) and 971 (85.1 %), respectively. The proportion of light drinkers was 30.1 % in all subjects.

**Table 1:** Subject characteristics

Number	1141
Age (years)	56.6 ± 8.3
BMI (kg/m^2^)	26.3 ± 3.6
WC (cm)	90.0 ± 7.2
SBP (mmHg)	131.1 ± 14.1
DBP (mmHg)	78.0 ± 9.4
Hypertension, *n* (%)	937 (82.1 %)
T-CHO (mg/dl)	212.2 ± 33.3
TG (mg/dl)	122.5 ± 62.8
HDL (mg/dl)	59.8 ± 13.8
LDL (mg/dl)	135.9 ± 30.1
Dyslipidemia, *n* (%)	1066 (93.4 %)
UA (mg/dl)	4.9 ± 1.1
FPG (mg/dl)	107.9 ± 19.0
HbA1c (NGSP) (%)	6.0 ± 0.7
IGT, *n* (%)	852 (74.7 %)
ALT (IU/l)	27.2 ± 19.4
AST (IU/l)	23.7 ± 10.8
GGT (IU/l)	34.1 ± 31.2
FIB-4 index	2.7 ± 0.7
Fatty liver, *n* (%)	642 (56.3 %)
V-type MS, *n* (%)	170 (14.9 %)
Light drinker, *n* (%)	344 (30.1 %)
Past history of smoking, *n* (%)	103 (9.0 %)

Data are given as means ± standard deviation (SD), and as number (%) for categorical variables

*ALT* alanine aminotransferase, *AST* aspartate aminotransferase, *BMI* body mass index, *DBP* diastolic blood pressure, *FPG* fasting plasma glucose, *GGT* gamma-glutamyl transpeptidase, *HbA1c* hemoglobin A1c, *HDL* high density lipoprotein, *IGT* impaired glucose tolerance, *LDL* low density lipoprotein, *MS* metabolic syndrome, *NGSP* National Glycohemoglobin Standardization Program, *SBP* systolic blood pressure, *T- CHO* total cholesterol, *TG* triglyceride, *UA* uric acid, *V-type* visceral type, *WC* waist circumference. Light drinkers were subjects who drank from more than zero to less than 20 g/day of alcohol

### Clinical characteristics in light drinkers and non-drinkers

The comparison of clinical characteristics in females with MS between light drinkers and non-drinkers is shown in Table 2. The *P*^a^ value was adjusted for confounding variables including age, BMI, WC, smoking history, dyslipidemia, hypertension, and IGT. Age, BMI, and WC were significantly lower in light drinkers than in non-drinkers. There was no significant difference in the prevalence of hypertension, dyslipidemia, and IGT between light drinkers and non-drinkers. HbA1c were significantly lower in light drinkers than in non-drinkers. Regarding liver enzyme, there was no significant difference in ALT or AST between light drinkers and non-drinkers. In addition, there was no significant difference in the prevalence of fatty liver or fatty liver with ALT elevation between light drinkers and non-drinkers.

**Table 2:** Comparison of clinical characteristics in females with MS between light drinkers and non-drinkers

	Light drinkers (*n* = 344)	Non-drinkers (*n* = 797)	*P*-value (*P* ^*a*^-value)
Age (years)	54.6 ± 7.9	57.5 ± 8.3	<0.001
BMI (kg/m^2^)	25.9 ± 3.5	26.4 ± 3.6	<0.05
WC (cm)	89.1 ± 7.1	90.3 ± 7.2	<0.05
SBP (mmHg)	129.7 ± 13.3	131.7 ± 14.4	(NS)
DBP (mmHg)	77.9 ± 9.3	78.0 ± 9.4	(NS)
Hypertension, *n* (%)	282 (82.0 %)	655 (82.2 %)	(NS)
T-CHO (mg/dl)	214.6 ± 34.8	211.1 ± 32.6	(NS)
TG (mg/dl)	121.5 ± 64.8	122.9 ± 62.0	(NS)
HDL (mg/dl)	60.2 ± 13.8	59.7 ± 13.8	(NS)
LDL (mg/dl)	138.0 ± 31.1	135.0 ± 29.6	(NS)
Dyslipidemia, *n* (%)	324 (99.4 %)	742 (93.1 %)	(NS)
UA (mg/dl)	4.9 ± 1.1	4.9 ± 1.1	(NS)
FPG (mg/dl)	106.5 ± 14.4	108.5 ± 20.6	(NS)
HbA1c (NGSP) (%)	5.9 ± 0.6	6.1 ± 0.8	(<0.05)
IGT, *n* (%)	261 (75.9 %)	591 (74.2 %)	(NS)
ALT (IU/l)	27.2 ± 21.4	27.2 ± 18.5	(NS)
AST (IU/l)	23.0 ± 9.9	24.0 ± 11.2	(NS)
GGT (IU/l)	32.3 ± 27.4	34.8 ± 32.6	(NS)
FIB-4 index	2.5 ± 0.6	2.7 ± 0.7	(NS)
Fatty liver, *n* (%)	188 (54.7 %)	454 (57.0 %)	(NS)
FL with ALT elevation, *n* (%)	76 (22.1 %)	177 (22.2 %)	(NS)
V-type MS, *n* (%)	55 (16.0 %)	115 (14.4 %)	(NS)
Past history of smoking, *n* (%)	49 (14.2 %)	54 (6.8 %)	<0.001

Data are given as means ± standard deviation (SD), and as number (%) for categorical variables. *P*-value is based on the *X*2 test or Student’s t-test. Significant is at the 5 % level

*ALT* alanine aminotransferase, *AST* aspartate aminotransferase, *BMI* body mass index, *DBP* diastolic blood pressure, *FL* fatty liver, *FPG* fasting plasma glucose, *GGT* gamma-glutamyl transpeptidase, *HbA1c* hemoglobin A1c, *HDL* high density lipoprotein, *IGT* impaired glucose tolerance, *LDL* low density lipoprotein, *MS* metabolic syndrome, *NGSP* National Glycohemoglobin Standardization Program, *NS* not significant, *SBP* systolic blood pressure, *T- CHO* total cholesterol, *TG* triglyceride, *UA* uric acid, *V-type* visceral type, *WC* waist circumference. ALT elevation was defined as ALT level of >31 IU/l in this study. Light drinkers were subjects who drank from more than zero to less than 20 g/day of alcohol. Non-drinkers were subjects who drank 12 drinks (one drink means less 10 g) or less per year of less than 10 g/drinking day

^a^Adjusted for age, BMI, WC, smoking history, dyslipidemia, hypertension, and IGT

### Clinical characteristics in minimal drinkers and very light drinkers

The comparison of clinical characteristics in females with MS between minimal drinkers and very light drinkers is shown in Table 3. The *P*^a^ value was adjusted for confounding variables including age, BMI, WC, smoking history, dyslipidemia, hypertension, and IGT. There was no significant difference in age, BMI, and WC between minimal drinkers and very light drinkers. SBP, DBP, and the prevalence of hypertension were significantly lower in minimal drinkers than in very light drinkers. HDL was significantly higher in very light drinkers than in minimal drinkers. HbA1c was significantly lower in very light drinkers than in minimal drinkers. The prevalence of fatty liver and ALT was significantly lower in very light drinkers than in minimal drinkers.

**Table 3:** Comparison of clinical characteristics in females with MS between minimal drinkers and very light drinkers

	Minimal drinkers (*n* = 250)	Very light drinkers (*n* = 94)	*P*-value (*P* ^*a*^-value)
Age (years)	54.6 ± 7.9	54.5 ± 7.8	NS
BMI (kg/m^2^)	25.8 ± 3.3	26.0 ± 4.0	NS
WC (cm)	89.0 ± 6.8	89.5 ± 8.0	NS
SBP (mmHg)	128.9 ± 13.4	131.8 ± 12.8	(<0.01)
DBP (mmHg)	77.3 ± 9.9	79.6 ± 7.5	(<0.01)
Hypertension, *n* (%)	197 (78.8 %)	85 (90.4 %)	(<0.001)
T-CHO (mg/dl)	213.2 ± 34.8	218.5 ± 34.5	(NS)
TG (mg/dl)	123.3 ± 67.7	116.6 ± 56.3	(NS)
HDL (mg/dl)	59.3 ± 13.9	62.8 ± 13.2	(<0.05)
LDL (mg/dl)	137.1 ± 31.0	140.3 ± 31.5	(NS)
Dyslipidemia, *n* (%)	235 (94.0 %)	89 (94.7 %)	(NS)
UA (mg/dl)	4.9 ± 1.1	5.1 ± 1.1	(NS)
FPG (mg/dl)	106.7 ± 14.7	105.9 ± 13.6	(NS)
HbA1c (NGSP) (%)	6.0 ± 0.6	5.8 ± 0.4	(<0.05)
IGT, *n* (%)	186 (74.4 %)	75 (79.8 %)	(NS)
ALT (IU/l)	28.5 ± 23.2	23.9 ± 15.2	(<0.05)
AST (IU/l)	23.0 ± 10.4	22.0 ± 8.0	(NS)
GGT (IU/l)	30.5 ± 22.9	36.9 ± 36.6	(NS)
FIB-4 index	2.5 ± 0.6	2.5 ± 0.6	(NS)
Fatty liver, *n* (%)	148 (59.2 %)	40 (42.6 %)	(<0.01)
FL with ALT elevation, *n* (%)	60 (24.0 %)	16 (17.0 %)	(NS)
V-type MS, *n* (%)	39 (15.6 %)	16 (17.0 %)	(NS)
Past history of smoking, *n* (%)	28 (11.2 %)	21 (22.3 %)	<0.05

Data are given as means ± standard deviation (SD), and as number (%) for categorical variables. *P*-value is based on the *X*2 test or Student’s t-test. Significant is at the 5 % level

*ALT* alanine aminotransferase, *AST* aspartate aminotransferase, *BMI* body mass index, *DBP* diastolic blood pressure, *FL* fatty liver, *FPG* fasting plasma glucose, *GGT* gamma-glutamyl transpeptidase, *HbA1c* hemoglobin A1c, *HDL* high density lipoprotein, *IGT* impaired glucose tolerance, *LDL* low density lipoprotein, *MS* metabolic syndrome, *NGSP* National Glycohemoglobin Standardization Program, *NS* not significant, *SBP* systolic blood pressure, *T- CHO* total cholesterol, *TG* triglyceride, *UA* uric acid, *V-type* visceral type, *WC* waist circumference. ALT elevation was defined as ALT level of >31 IU/l in this study. Minimal drinkers were subjects who drank from more than zero to less than 10 g/day of alcohol. Very light drinkers were subjects who drank from 10 to less than 20 g/day of alcohol

^a^Adjusted for age, BMI, WC, smoking history, dyslipidemia, hypertension, and IGT

### Treatment for lifestyle-related diseases in light drinkers and non-drinkers

The comparison of those receiving treatment for lifestyle-related diseases between light drinkers and non-drinkers in females with MS is shown in Table 4. The *P*^a^ value was adjusted for confounding variables including age, BMI, WC, smoking history, dyslipidemia, hypertension, and IGT. There was no significant difference in the prevalence of those receiving treatment for hypertension, SBP, or DBP between light drinkers and non-drinkers. The prevalence of those receiving treatment for dyslipidemia was significantly lower in light drinkers than in non-drinkers. The prevalence of those receiving treatment for IGT or FBS in those receiving treatment for IGT was significantly lower in light drinkers than in non-drinkers.

**Table 4:** Comparison of those receiving treatment for lifestyle-related diseases between light drinkers and non-drinkers in females with MS

Lifestyle-related disease	Presence of treatment	Light drinkers (*n* = 344)	Non-drinkers (*n* = 797)	*P*-value (*P* ^a^-value)
Hypertension	Treatment (+), *n* (%)	128 (37.2 %)	330 (41.4 %)	NS
	SBP (mmHg)	130.5 ± 13.7	133.2 ± 15.7	(NS)
	DBP (mmHg)	77.3 ± 8.6	78.6 ± 9.2	(NS)
	Treatment (−), *n* (%)	216 (62.8 %)	467 (58.6 %)	
	SBP (mmHg)	129.2 ± 13.0	130.6 ± 13.4	(NS)
	DBP (mmHg)	78.3 ± 9.7	77.6 ± 9.6	(NS)
Dyslipidemia	Treatment (+), *n* (%)	85 (24.7 %)	320 (40.2 %)	<0.01
	T-CHO (mg/dl)	197.8 ± 32.4	201.0 ± 26.5	(NS)
	TG (mg/dl)	116.3 ± 49.4	113.4 ± 52.6	(NS)
	HDL (mg/dl)	63.8 ± 13.8	63.0 ± 14.0	(NS)
	LDL (mg/dl)	119.2 ± 26.3	122.7 ± 23.4	(NS)
	Treatment (−), *n* (%)	259 (75.3 %)	477 (59.8 %)	
	T-CHO (mg/dl)	220.2 ± 33.8	218.0 ± 34.5	(NS)
	TG (mg/dl)	123.2 ± 69.1	129.3 ± 66.9	(NS)
	HDL (mg/dl)	59.1 ± 13.6	57.4 ± 13.1	(NS)
	LDL (mg/dl)	144.2 ± 30.1	143.3 ± 30.5	(NS)
IGT	Treatment (+), *n* (%)	17 (4.9 %)	72 (9.0 %)	<0.05
	FBS (mg/dl)	124.1 ± 25.4	138.1 ± 33.4	(<0.05)
	HbA1c (NGSP) (%)	6.9 ± 0.9	7.2 ± 1.1	(NS)
	Treatment (−), *n* (%)	327 (95.1 %)	725 (91.0 %)	
	FBS (mg/dl)	105.6 ± 13.0	105.5 ± 16.2	(NS)
	HbA1c (NGSP) (%)	5.9 ± 0.5	6.0 ± 0.7	(NS)

Data are given as means ± standard deviation (SD), and as number (%) for categorical variables. *P*-value is based on the *x*2 test or Student’s t-test. Significant is at the 5 % level

*DBP* diastolic blood pressure, *FPG* fasting plasma glucose, *HbA1c* hemoglobin A1c, *HDL* high density lipoprotein, *IGT* impaired glucose tolerance, *LDL* low density lipoprotein, *NGSP* National Glycohemoglobin Standardization Program, *NS* not significant, *SBP* systolic blood pressure, *T-CHO* total cholesterol, *TG* triglyceride, Light drinkers were subjects who drank from more than zero to less than 20 g/day of alcohol. Non-drinkers were subjects who drank 12 drinks (one drink means less 10 g) or less per year ofless than 10 g/drinking day

^a^Adjusted for age, BMI, WC, smoking history, dyslipidemia, hypertension, and IGT

### Predictors of fatty liver with ALT elevation

Univariate and multivariate independent predictors of fatty liver with ALT elevation in females with MS are shown in Table 5. Of the 20 items related to the clinical background of subjects with or without fatty liver with ALT elevation, 14 items were identified as significant factors by univariate analysis. Multiple logistic regression analysis was performed with 12 items, with age, BMI, WC, SBP, DBP, TG, HDL, UA, IGT, MS type, drinking status, and smoking status as covariates. BMI, WC, DBP, TG, UA, IGT, and V-type MS were significant and independent predictors of an increased prevalence of fatty liver with ALT elevation. The odds ratio [OR] (95 % confidence interval [CI], *p* value) for fatty liver with ALT elevation were as follows: BMI, 2.181 (1.445–3.293, *p* <0.001); WC 1.853 (1.280–2.684, *p* <0.01); DBP 1.604 (1.120–2.298, *p* <0.05); TG 2.202 (1.562–3.105, *p* <0.001); UA 2.959 (1.537–5.698, *p* <0.01); IGT 1.692 (1.143–2.506, *p* <0.01); and V-type MS 3.708 (2.529–5.437, *p* <0.001).

**Table 5:** Results of univariate and multivariate: independent predictors of fatty liver with ALT elevation in females with MS

	Univariate analysis			Multivariate analysis		
	FL with ALT elevation (+) (*n* = 253)	FL with ALT elevation (−) (*n* = 888)	*P*-value	OR	95 % CI	*P*-value
Age (≧50/< 50 years)	187/66	739/149	<0.01	1.003	0.677–1.484	NS
BMI (≧25/< 25 kg/m^2^)	199/54	469/419	<0.001	2.181	1.445–3.293	<0.001
WC (≧90/< 90 cm)	166/87	348/540	<0.001	1.853	1.280–2.684	<0.01
SBP (≧130/< 130 mmHg)	138/115	557/331	<0.05	0.756	0.543–1.053	NS
DBP (≧85/< 85 mmHg)	76/177	197/691	<0.05	1.604	1.120–2.298	<0.05
Hypertension (+/−)	209/44	728/160	NS			
T-CHO (≧220/< 220 mg/dl)	103/150	340/548	NS			
TG (≧150/< 150 mg/dl)	105/148	206/682	<0.001	2.202	1.562–3.105	<0.001
HDL (<50/≧ 50 mg/dl)	96/157	217/671	<0.001	1.362	0.955–1.942	NS
LDL (≧140/< 140 mg/dl)	118/135	365/523	NS			
Dyslipidemia (+/−)	236/17	58/830	NS			
UA (≧7.0/< 7.0 mg/dl)	24/229	23/865	<0.001	2.959	1.537–5.698	<0.01
FPG (≧100/< 100 mg/dl)	202/51	641/247	<0.05			
HbA1c (≧6.5/< 6.5 % NGSP)	70/183	110/778	<0.001			
IGT (+/−)	204/49	648/240	<0.05	1.692	1.143–2.506	<0.01
AST (≧31/< 31 IU/l)	140/113	21/867	<0.001			
GGT (≧51/< 51 IU/l)	90/163	94/794	<0.001			
V-type MS/S-type MS	82/171	88/800	<0.001	3.708	2.529–5.437	<0.001
Light drinker/Non-drinker	76/177	268/620	NS	0.988	0.699–1.396	NS
Past history of smoking (+/−)	31/222	72/816	NS	1.182	0.705–1.981	NS

Significant is at the 5 % level

*ALT* alanine aminotransferase, *AST* aspartate aminotransferase, *BMI* body mass index, *CI* confidence interval, *DBP* diastolic blood pressure, *FL* fatty liver, *FPG* fasting plasma glucose, *GGT* gamma-glutamyl transpeptidase, *HbA1c* hemoglobin A1c, *HDL* high density lipoprotein, *IGT* impaired glucose tolerance, *LDL* low density lipoprotein, *MS* metabolic syndrome, *NGSP* National Glycohemoglobin Standardization Program, *NS* not significant, *OR* odds ratio, *SBP* systolic blood pressure, *S-type* subcutaneous-type, *T-CHO* total cholesterol, *TG* triglyceride, *UA* uric acid, *V-type* visceral type, *WC* waist circumference. Light drinkers were subjects who drank from more than zero to less than 20 g/day of alcohol. Non-drinkers were subjects who drank 12 drinks (one drink means less 10 g) or less per year of less than 10 g/drinking day. ALT elevation was defined as ALT level of >31 IU/l in this study

## Discussion

This cross-sectional study explored the association between LAC and fatty liver with ALT elevation in females with MS. The association between MS and NAFLD has been shown to be mutually very strong [13, 14], and NAFLD is thought to be a manifestation of MS in the liver [13, 15, 16]. Light or moderate drinking has been reported to play a protective role against fatty liver in several studies [4–8]. Moreover, a recent study showed that the prevalence of NAFLD was significantly lower in light drinkers than in non-drinkers, and LAC was one of the significant factors of decreased prevalence of NAFLD, including males with MS [9]. However, the prevalence of MS, NAFLD including fatty liver, and the influence of alcohol consumption has been known to differ markedly between males and females. Additionally, females have been known to be more sensitive to the hepatic effects of alcohol than males in clinical and experimental studies [17–19]. However, the association between LAC and fatty liver with ALT elevation in females with MS is unclear. Results from this study demonstrated that there was no significant difference in the prevalence of fatty liver with ALT elevation between light drinkers and non-drinkers; however, the prevalence of those undergoing treatment for dyslipidemia and IGT was significantly lower in light drinkers than in non-drinkers in females with MS. BMI, WC, V-type MS, and lifestyle-related disease were important factors for the prevalence of fatty liver with ALT elevation in females with MS.

Ethanol induces hepatic lipid synthesis and may reduce hepatic fatty acid oxidation [20]. However alcohol consumption is known to cause fatty liver in some cases [20, 21]. Light or moderate drinking was associated with a lower incidence of hypertransaminasemia [5, 6] and a decreased risk of fatty liver [4, 7, 8] in recent clinical studies. The prevalence of fatty liver was reported to be significantly decreased by light or moderate drinking in a study with asymptomatic Japanese male subjects [7]. However, the influence of alcohol intake is known to differ between males and females [17–19, 22], and the association between liver enzyme, NAFLD including fatty liver, and LAC in females has been controversial. It was reported that the odds ratio for MS was decreased to less than 1 in females with LAC [23], and the prevalence of fatty liver in females was reported to be significantly lower in drinkers than in non-drinkers [8]. On the other hand, it was reported in a recent study that there was no association between the prevalence of elevated liver enzyme levels and alcohol consumption in females [24]. Additionally, in a recent prospective study from the UK, excess body weight was reported to contribute to almost 20 % of liver cirrhosis-related hospital admission and deaths in middle-aged female subjects, and alcohol consumption contributed to almost 50 % [25]. However, in the majority of reports on the effect of alcohol consumption on MS, subjects were almost always from a general population and were not divided into those with and without MS, or those with fatty liver were not divided with respect to liver enzyme elevation. To address this, the present study was limited to only female subjects with MS, and showed that there was no significant difference in the prevalence of fatty liver with ALT elevation between light drinkers and non-drinkers.

The mechanism of this inverse association between drinking and NAFLD including fatty liver remains unclear. Light to moderate drinking may enhance insulin sensitivity [21, 26] and reduce the risk of non-alcoholic steatohepatitis, possibly due to reduced insulin resistance [4]. Although the present study showed that there was no significant difference in the prevalence of fatty liver with ALT elevation or IGT between light drinkers and non-drinkers, the prevalence of those undergoing treatment for IGT was significantly lower in light drinkers than in non-drinkers in females with MS. It is known that obesity increases fat deposition in hepatocytes and the progression of fatty liver, which may lead to inflammation, liver fibrosis, and liver cirrhosis [27]. The improvement of insulin sensitivity by light drinking may have been weak because subjects in the present study were obese with MS. The beneficial effects of reducing a risk of fatty liver may be weak in light female drinkers with MS, due to the lack of significant difference in insulin sensitivity between light drinkers and non-drinkers in females with MS.

Although it was reported that drinking had a positive effect on HDL-C levels [28], there was no significant difference in HDL levels between light drinkers and non-drinkers in females with MS in the present study. Interestingly, HDL was significantly higher in very light drinkers than in minimal drinkers. The effect of alcohol consumption on MS in the general population remains controversial. Although several reports have shown that the prevalence of MS is associated with alcohol consumption, irrespective of the amount consumed [29, 30], some another studies have reported beneficial effects of alcohol consumption on MS [31, 32]. It was recently reported that the prevalence of fatty liver was decreased along with the level of alcohol consumption in males subjects with MS, and alcohol consumption was associated with higher blood pressure, higher fasting plasma glucose, and a lower level of HDL-C in the male subjects who drank from more than zero to an excess of 280 g/week [23]. Although the present study showed that there was no significant difference in the prevalence of fatty liver with ALT elevation, hypertension, dyslipidemia, or IGT between light drinkers and non-drinkers, the prevalence of those undergoing treatment for dyslipidemia or IGT was significantly higher in non-drinkers than light drinkers. These results suggest that females may differ from males in sensitivity to the hepatic effects of alcohol due to some factors, such as pharmacokinetics or metabolism of alcohol [18]. Additionally, the present results may also be influenced by the difference in adipose tissue distribution [33, 34], estrogen-related sex hormones [35, 36], treatment for lifestyle-related disease, and other social lifestyle factors, such as physical activity, exercise, diet, and drinking patterns between males and females. Further investigation is warranted, due to the lack of more detailed investigation on social lifestyle factors.

We do not suggest that drinking is recommended for non-drinkers because LAC was not a significant factor of a decreased prevalence of fatty liver with ALT elevation in the present study, and the effects of drinking differ among individuals. Increased drinking is also not recommended for minimal drinkers. Although ALT and the prevalence of fatty liver were significantly lower in very light drinkers than in minimal drinkers, the prevalence of hypertension was significantly higher in very light drinkers than in minimal drinkers. Alanine aminotransferase and lifestyle-related diseases, such as hypertension, dyslipidemia, and IGT, may be more affected in non-drinkers and minimal drinkers with MS by the increased eating duration due to alcohol consumption. Interestingly, the prevalence of hypertension in light daily drinkers (from more than zero to less than 10 g/every day) was shown to be significantly higher than in non-drinkers in the present study (data not shown). In addition, individuals with MS and patients with risk of drinking abuse should not be advised to drink heavily. In particular, heavy drinking should not be recommended for individuals with MS because excess alcohol consumption is known to be a risk for mortality and death from noncardiovascular causes [37, 38], and MS is known to be a risk factor for arteriosclerotic diseases and ischemic heart disease [39]. Despite the possibility that heavy drinking may have a beneficial effect against fatty liver and on liver enzyme, we emphasize that heavy drinking should not be recommended for individuals with MS.

There are some limitations that should be noted in the present study. First the present study did not investigate the underlying mechanism between the association of LAC and fatty liver with ALT elevation in females with MS, due to the nature of the cross-sectional design. Second, detailed data, such as contents of subjects’ diets, eating duration, total caloric intake, and physical activity were not investigated. Third, although the subjects in the present study were females, the state of menses was not investigated, and the levels of estrogen-related sex hormones were not measured. Fourth, there was a possibility that the proportion of those with mild steatosis undetectable by ultrasound may have been higher in light drinking subjects than in non-drinkers, because the sensitivity of detecting steatosis by ultrasound is known to be below 20–30 %. Fifth, although ALT is known to be a simple marker of liver enzymes, ALT is unable to reflect the severity of NAFLD. Last, there was a possibility of selection bias subjects in the present study may have high interest in health because they hoped to undergo a medical check-up in Ningen Dock. Further studies will be required to resolve these limitations.

## Conclusion

The present study showed that there was no significant difference in the prevalence of fatty liver with ALT elevation between light drinkers and non-drinkers in females with MS. Body mass index, WC, DBP, TG, UA, IGT, and V-type MS were significant factors of an increased prevalence of fatty liver with ALT elevation, and the prevalence of individuals undergoing treatment for dyslipidemia or IGT was significantly lower in light drinkers than in non-drinkers in females with MS. We believe that BMI, WC, V-type MS, and lifestyle-related disease may be more important factors for the prevalence of fatty liver with ALT elevation than LAC in Japanese females with MS.

## Abbreviations

AFI: abdominal wall fat index
ALT: alanine aminotransferase
AST: aspartate aminotransferase
BMI: body mass index
CI: confidence interval
DBP: diastolic blood pressure
DM: diabetic mellitus
FPG: fasting plasma glucose
GGT: gamma-glutamyl transpeptidase
HbA1c: hemoglobin A1c
HBsAg: hepatitis B surface antigen
HCVAb: hepatitis C antibody
HDL: high-density lipoprotein
IDF: International Diabetes Federation
IGT: impaired glucose tolerance
LAC: light alcohol consumption
LDL: low-density lipoprotein
MS: metabolic syndrome
NAFLD: non-alcoholic fatty liver disease
NGSP: National Glycohemoglobin Standardization Program
NS: not significant
OR: odds ratio
SBP: systolic blood pressure
SD: standard deviation
S-type: subcutaneous type
T-CHO: total cholesterol
TG: triglyceride
UA: uric acid
V-type: visceral type
WC: waist circumference
